# The Wear Rate Forecast of MgO-C Materials Type MC95/10 in the Slag Spout Zone of an Oxygen Converter in Terms of the Bayesian Estimation

**DOI:** 10.3390/ma15093065

**Published:** 2022-04-22

**Authors:** Wiesław Zelik, Sebastian Sado, Ryszard Lech

**Affiliations:** 1 Research and Development Centre of Ceramics, Zakłady Magnezytowe “ROPCZYCE” S.A., ul. Postępu 15c, 02-676 Warszawa, Poland; wieslaw.zelik@ropczyce.com.pl; 2 Faculty of Materials Science and Ceramics, AGH University of Science and Technology, al. Mickiewicza 30, 30-059 Kraków, Poland; rysz.lech@gmail.com

**Keywords:** oxygen converter, refractories, wear forecasting, Bayesian statistics

## Abstract

The ceramic–carbon refractory lining of an oxygen converter is subjected to variable thermochemical stresses, causing a progressive loss of material over time, which is expressed in a decreasing residual thickness of the lining. The forecasting method using Bayesian statistics has become a valuable skill in steel production planning and is one of the main conditions constituting the appropriate organization of steel and refractories production. This paper presents examples of Bayesian modelling of the unit wear rate value of the refractory materials for the zone with the highest wear in the refractory lining of a converter. From the experience gained during long-term operation of a steel-producing oxygen converter, it was found that the value of the unit wear rate of the refractory material in the slag spout zone of the steel-producing oxygen converter is subjected to an *a posteriori* normal distribution, with the following parameters: mean value µ = 401.23 µ heat^−1^, standard deviation σ = 13.74 µm heat^−1^. The forecasted mean value of the unit wear rate of the MC95/10 refractories lined in the slag spout zone of the oxygen converter used for steel production, and which operates in intensive exploitation conditions, was equal to µ = 420 µm heat^−1^.

## 1. Introduction

The oxygen converter lined with MgO-C refractory materials is one of the two main units used to prepare ferroalloy for secondary metallurgy processing. It is estimated that the amount of steel produced by the converter process is currently about 50% of heated steel [1].

The high process temperatures, changing chemical composition of the slag, and turbulent flow of the heterophase metallic-ceramic liquid caused by blowing oxygen into it, make the working conditions of the refractories in the oxygen converter very variable. The wear process of refractories is dominated by the cyclic processes of oxidation of the carbon part of MgO-C type refractories, dissolution, and abrasion of the near-surface parts of materials, caused by penetration of the liquid alloy [2,3].

The problem of modeling the wear of the refractory materials under the influence of different metallurgical factors has been discussed by various authors [4,5,6,7,8].

**Figure 1:**
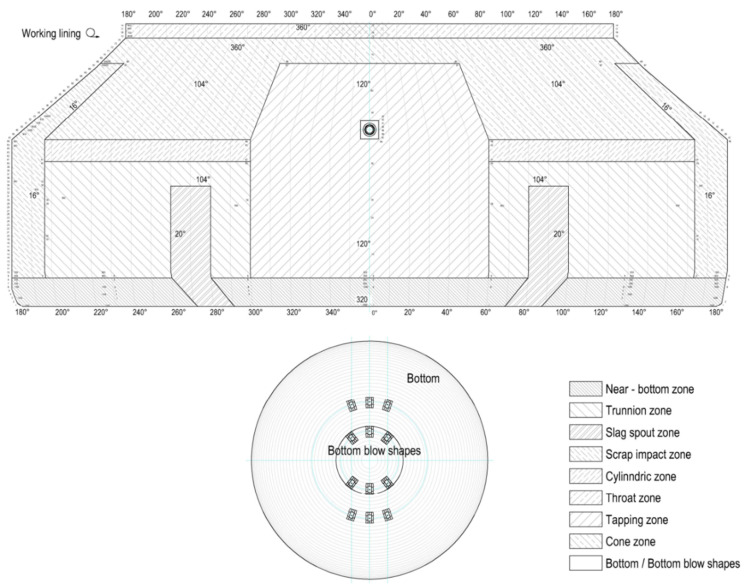
Zone scheme of the working lining with refractory materials.

In oxygen converters, intensive oxidation of the carbon, silicon, phosphorus, and manganese contained in the batch results in uneven wear of the refractory lining. For this reason, refractories are lined in the converter in a zoned manner, as shown in Figure 1. In the refractory lining zones with particularly unfavorable effects from corrosive factors, the best quality refractories are used. The greatest wear zone of the refractory lining in the oxygen converter is the so-called slag spout zone. The slag spout zone consists of two areas in the converter (left and right trunnion), lying symmetrically in relation to the taphole and with difficult access for maintenance of the refractory lining. 

In the slag spout zone, the thermal and chemical loads are mainly concentrated during the converter campaign. An exception is the initial stage, which usually lasts until about the hundredth heat, where thermomechanical loads caused by thermal stresses generally dominate. 

The purpose of the paper is to present the possibility of applying Bayesian modelling in predicting the ranges of reliability of the unit wear rate of the refractory oxygen converter lining in the slag spout zone during the converter campaign. At the same time, the assumed values of the *a priori* distribution of the wear rate value result from the known metallurgical working conditions, to which the MgO-C material of the MC 95/10 type is exposed to. The result of the calculations presented in the article is the *a posteriori* distribution of the wear rate, from which the desired range of reliability of this rate, treated in the calculations as a random variable, is calculated. This paper also gives a procedure for predicting the unit wear rate value of the refractory lining in the slag spout zone of an oxygen converter.

## 2. Materials and Methods

The subject of the study is unit wear rate of the spout refractory lining of an oxygen converter type TBM (Thyssen bottom metallurgy), with a nominal load capacity of 350 tons. The converter completed 2063 heats and was shut down due to the wear of the spout zone’s refractory lining. The converter was operated from August 2018 to January 2019. A high purity MgO-C material bonded with pitch was used in the slag spout zone. The material is classified according to PN-EN ISO 10081-3:2006 (classification of dense shaped refractory products—Part 3: basic products containing from 7 percent to 50 percent residual carbon) as type MC 95/10. Its chemical composition determined by WD XRF technique based on PN-EN ISO 26845:2009 (Chemical analysis of Refractory products by XRF—Fused cast bead method) is presented in Table 1.

Shaped bricks made of the refractory material were formed on a hydraulic press with a unit pressure of over 1200 bar. The slag spout zone was lined with a combination of 90/80 and 90/20 flat wedges. The dimensions of the wedges are: 900 × 100 × 190/110 mm and 900 × 100 × 160/140 mm. The initial thickness of the working lining in the slag spout zone was 900 mm.

The converter is not equipped with a slag splashing installation. During the campaign, however, the refractory lining of the converter was maintained by gunning and slagging. The chemical compositions of the slags were typical for the converter process. Spectrometric, oxide chemical analysis of samples collected (*n* = 1459) during the campaign showed an average content of MgO, CaO, SiO_2_, Fe_2_O_3_, Al_2_O_3_ of 6.33 wt.%, 46.85 wt.%, 13.22 wt.%, 15.67 wt.%, 1.26 wt.%, respectively. The average basicity of the slag was about 3.74.

The wear rate of a refractory lining is usually expressed by the material wear rate per heat. The material loss depends on many process factors and on the properties of the lining material in a given zone. The unit wear rate of refractory materials is the basis for assessing the economic efficiency of the used materials, measured by the unit cost of steel production. The result of the lining thickness control, especially in the final stage of the converter operation, is the basis of the decision to end the equipment’s operation. On the basis of the measurements made during the campaign, decisions are also made on gunning and/or slagging the working lining of the oxygen converter.

Measurements of the residual thickness of the lining are made with a laser device. The device is placed in front of the empty converter in the slag spout position, so that a concentrated photon beam can scan the inside of the device. Before starting the converter campaign, a baseline measurement of the refractory lining is taken as a reference for the residual thickness measurements of the refractory working lining. The baseline measurement for subsequent measurements of the residual working thickness of the lining is taken at the converter’s safety lining. The safety lining is installed behind the working layer and provides refractory protection of the converter against liquid steel leakage when the working lining is already heavily worn. Safety linings are usually made of burnt magnesia materials type M90 or M95 (PN-EN ISO 10081-2:2006 (classification of dense shaped refractory products—Part 2: basic products containing less than 7% residual carbon). The frequency of the residual thickness measurements is dependent on the availability of the device (converter) and the condition of its refractory working lining. The results of the measurements, in the form of wear maps with values of residual thickness in all zones, constitute the basis for assessment of the condition of the refractory lining, qualifying the converter for further operation. The unit wear rate of the refractory materials is calculated from the residual thicknesses measured during the converter’s campaign, as the difference of the consecutively measured residual thicknesses of the refractory lining related to the difference in the number of heats, and dividing these measurements according to the formula:

w_i_ = (G_k_ − G_i_)/(i − k) (1)

where w_i_—unit wear rate, G—laser-measured residual thickness, i and k are the number of successive heats for which measurements have been made, where i > k.

In the calculation part of the paper, Bayesian inference was used. This allows the use of *a priori* information (often called initial belief), resulting from knowledge of the research problem and the knowledge of the given phenomenon, in order to obtain *a posteriori* information (often called empirical probability, outcome), obtained using the likelihood function. This methodology is based on Bayes’ theorem of conditional probability, known from probability calculus [9,10,11]:

(2) $$P\left(A|B\right)=\frac{P\left(B|A\right)P\left(A\right)}{P\left(B\right)}$$

where P(A|B), P(B|A) are conditional probabilities, and P(A), P(B) are probabilities of events A and B. Bayes’ theorem for a continuous probability distribution has the form [12]:

(3) $$P\left(\mu |\mathrm{dane}\right)=\frac{P\left(\mathrm{dane}|\mu \right)\cdot P\left(\mu \right)}{\int_{-\infty }^{\infty }P\left(\mathrm{dane}|\mu \right)\cdot P\left(\mu \right)d\mu }$$

The derivation of the formulas for the mean value and parameters’ precision of the a *posteriori* distribution when the *a priori* distribution is a normal distribution, and the observed data obtained from a normal distribution is given, for example, in [6]. These quantities are expressed by Equations (4) and (5):

(4) $$\mu_{\mathrm{aposteriori}}=\frac{\tau_{0}\cdot \mu_{0}+\tau \cdot \sum_{i=1}^{n}w_{i}}{\tau_{0}+n\cdot \tau }$$

(5) $$\tau_{\mathrm{aposteriori}}=\tau_{0}+n\cdot \tau$$

where $\tau =1/\sigma^{2}$ is the precision, σ—standard deviation, n—number of measurements of analyzed quantity, $w_{i}$—consecutive measurement, τ_0_—assumed precision of the distribution, indices zero denote hyperparameters of the distribution that result from *a priori* knowledge of the problem. In another notation, without the use of the quantity called precision, formulas for the mean value and variance of *a posteriori* distribution are derived, e.g., in the work [13].

## 3. Results

**Figure 2:**
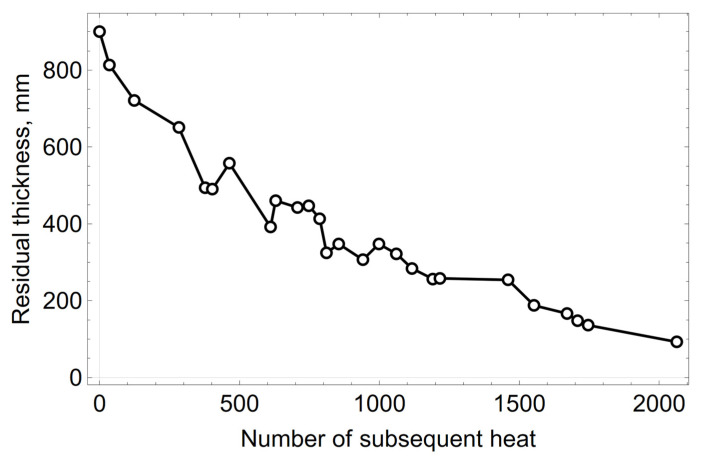
Average residual thickness of the refractory lining of the spout zone.

The averaged results of the laser measurements of the wear of the oxygen converter’s refractory lining in the slag spout zone (left and right spout) are shown in Figure 2.

**Figure 3:**
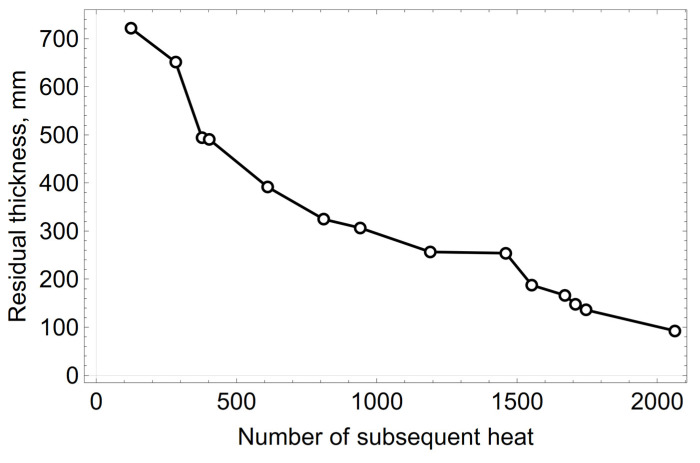
Corrected average residual thickness of the refractory lining of the spout zone.

The thickness of the refractory lining of the converter decreases over time. The points visible in Figure 2, where an increase in the thickness of the lining was found, come from measurements taken after maintenance (repair) of the converter’s refractory lining, using auxiliary materials. This means that, in order to protect the refractory lining, a layer of material, in the form of refractory gunning masses and/or processed slag partially saturated with MgO, had been applied to the walls of the converter, which reduces the wear rate of the working lining. In the following calculations, the thickness measurements of the refractory lining for which an increasing residual thickness was observed compared to the previous measurement were not considered. Measurements made at the beginning of the campaign were also not taken into account. The high loss of the refractory lining, expressed by the loss of the refractory material in the slag spout zone, amounted to 182 mm in the initial period of the converter operation, till the 123th heat. As already mentioned, this was connected with thermomechanical loads. Therefore, for further calculations the measured data from the 124th heat and onwards were adopted. The corrected curve of the residual thickness measurements is shown in Figure 3.

**Figure 4:**
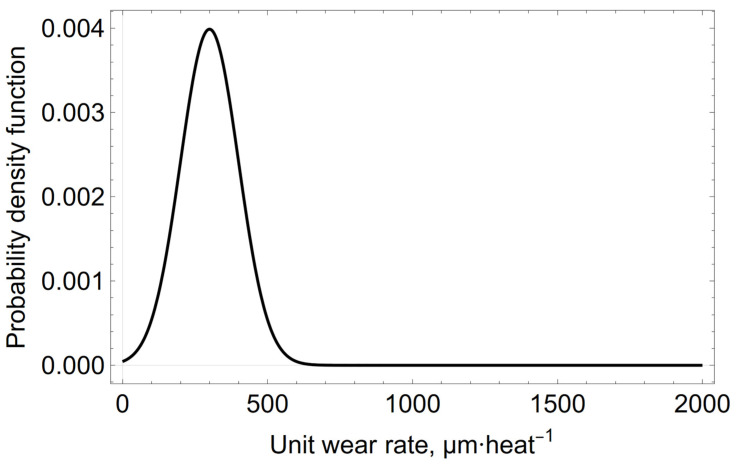
*A priori* normal distribution, expressing the initial belief in the distribution of the unit wear rate with parameters μ_0_ = 300 µm heat^−1^, σ_0_ = 100 µm heat^−1^.

The wear rate values of the spout zone’s refractory lining for the cases considered in the graph shown in Figure 3 are as follows: w_n_ = 443, 1670, 135, 476, 335, 138, 200, 9, 715, 179, 487, 316, and 137 µm heat^−1^, where n = 1, …, 13. From the data analysis of the previous converter campaigns, it is believed that the average wear rate in the campaign will be μ_0_ = 300 µm heat^−1^, with the standard deviation σ_0_ = 100 µm heat^−1^. The *a priori* distribution of the unit wear rate values of the refractory lining expressed by the normal distribution for the assumed assumptions is presented in Figure 4.

**Figure 5:**
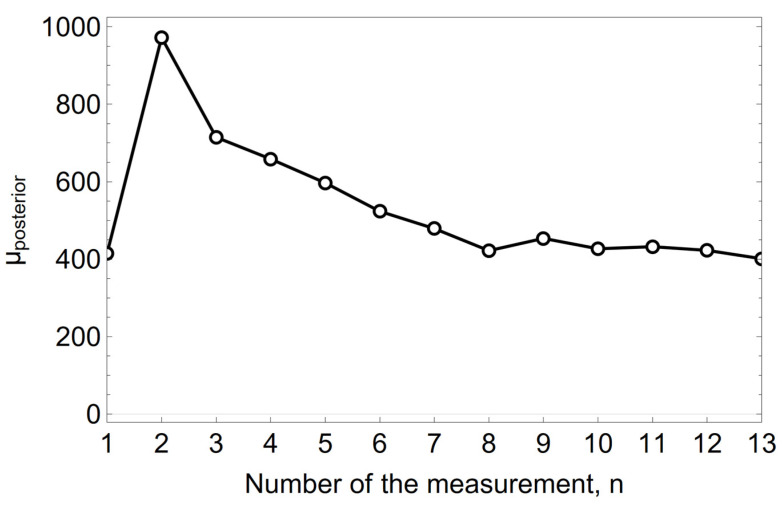
The variation of the mean value of μ µm heat^—1^ of the *a posteriori* distribution of the unit refractory wear rate in the spout zone.

**Figure 6:**
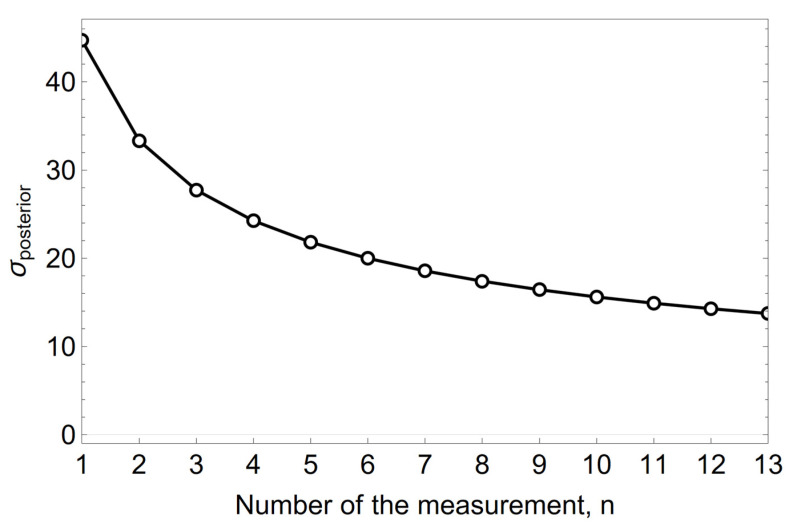
The variation of the standard deviation σ µm heat^−1^ of the *a posteriori* distribution of the unit refractory wear rate in the spout zone.

The mean value of the *a posteriori* normal distribution for the conjugate solution, assuming the normality of the *a priori* distribution, is calculated according to Formulas (1) and (4). The precision, which is the inverse of the variance σ^2^, is expressed by Formula (5). For a known standard deviation σ = 50 µm heat^−1^ and with the hyperparameters of the *a priori* distribution being μ_0_ = 300 µm heat^−1^ and *σ*_0_ = 100 µm heat^−1^, the values of μ_aposteriori_ and τ_aposteriori_ calculated from Formulas (4) and (5) for successive measurements are shown in Figure 5 and Figure 6.

**Figure 7:**
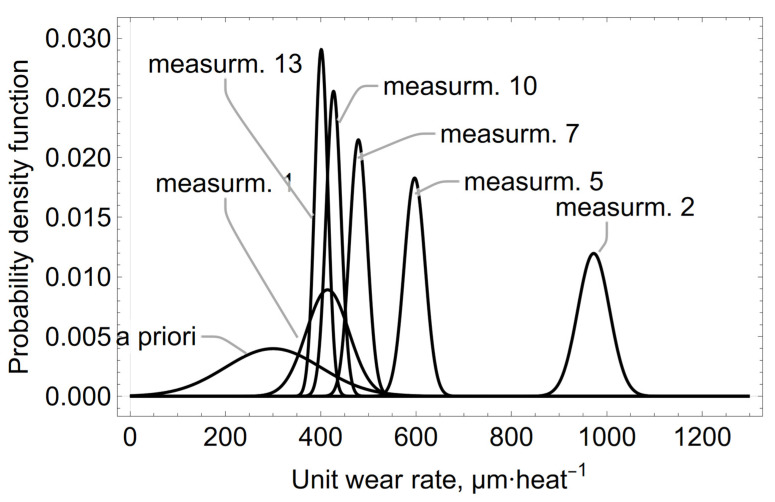
The Bayesian evolution of the mean value of μ µm heat^−1^ unit wear rate of MC 95/10 refractory material in the spout zone, calculated for selected measurements: 1, 2, 5, 7, 10, 13.

The evolution of the *a posteriori* distribution of the average refractory wear rate of the spout zone, calculated from Equations (4) and (5) for 13 consecutive rate measurements, is shown in Figure 7.

The obtained results allow the calculation of credible intervals for the random variable µm heat^−1^, which are equivalent to the confidence intervals in the frequency approach to statistical inference. Thus, the average unit wear rate of refractories after 13 measurements is μ = 401.23 µm heat^−1^, standard deviation σ = 13.74 µm heat^−1^. The calculated quantiles of the unit wear rate for *a posteriori* distribution are shown in Table 2.

The resulting *a posteriori* distribution for the Bayesian inference can be used as a starting point for further calculations, e.g., to forecast the wear rate. With the new results, a new confidence interval can be calculated, as well as a new mean value and standard deviation of the *a posteriori* distribution.

The calculations above were carried out using conjugated solutions, where the *a priori* distribution and the likelihood function were expressed by normal distributions. The result of the calculation, in the form of an *a posteriori* probability distribution of the unit refractory wear rate value, also belongs to the class of normal probability distributions of random variables.

These calculations were verified using the R software and one of the libraries supporting Bayesian inference. In contrast to the conjugated solution, the use of the Monte Carlo method based on Markov chains involves the generation of random samples from *a posteriori* distributions, together with the approximation of their characteristics. The results obtained using the MCMCpack library and the Monte Carlo algorithm [14,15,16], sampling in a Markov chains sequence for 10,000 samples, can be obtained using the following R code:
library(MCMCpack) #data w = c(443, 1670, 135, 476, 335, 138, 200, 9, 715, 179, 487, 316, 137) #known standard deviation sigma2 = 50^2 # hyperparameters of the apriori distribution mu0 = 300 tau20 = 100^2 # function call to calculate the distribution of aposteriori set.seed(1234) posterior <− MCnormalnormal(y, sigma2 = sigma2, mu0 = mu0, tau20 = tau20, 10,000) # summary of results summary(posterior) quantile(posterior, c(0.025,0.5,0.975)) mean(posterior) # apriori and aposteriori distribution diagram plot(posterior) grid <- seq(0,1300,0.5) plot(grid, dnorm(grid, 300, 100), type = “l”, col = “red”, lwd = 3, ylim = c(0,0.03), xlab = “mu”, ylab = “density”) lines(density(posterior), col = “blue”, lwd = 3) legend(‘topright’, c(“prior”, “posterior”), lwd = 3, col = c(“red”, “blue”))

The results obtained using the R code and the MCMCpack library are:

- average wear rate of 401.22 µm heat^−1^,
- 95% confidence interval: [374.77, 427.86] µm heat^−1^.

The accuracy of the calculations was also checked using the “MetropolisHastingsSequence” function [17] available in the repository of additional functions of the Wolfram language [18]. The calculations performed with the use of Wolfram Mathematica version 12.31 allowed obtaining the 95% confidence interval of the unit refractory wear rate in the slag spout zone of the converter, being:

375.7 ≤ µ_aposterior_ ≤ 428.2 µm heat^−1^. (6)

Both ranges are insignificantly different.

The unit wear rate value of the above described refractory lining of the oxygen converter treated as a random variable was experimentally verified after the oxygen converter’s campaigns, which were in operation between February 2019 and November 2019. The mean value of the unit wear rate was calculated as µ = 420 µm heat^−1^, and this is within the above calculated confidence ranges.

**Figure 8:**
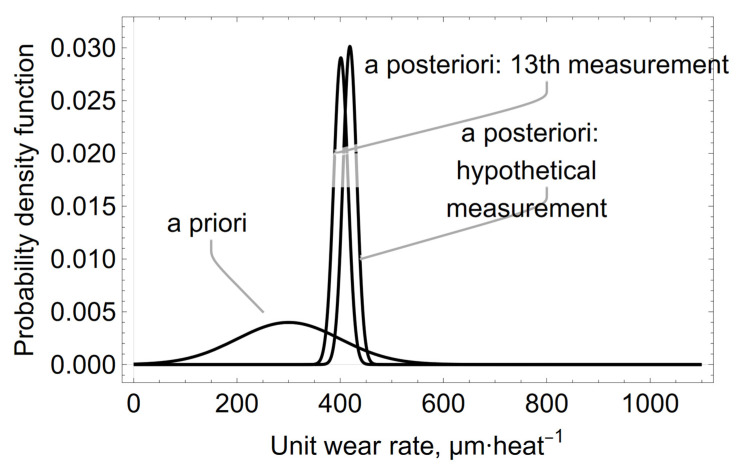
The prediction of the *a posteriori* distribution for the unit wear rate calculated for the hypothetical 14th measurement of *x_p_* = 650 µm heat^−1^.

In order to predict the value of the unit wear rate of the refractory lining in the slag spout zone of the oxygen converter, it was assumed that the hypothetical 14th measurement result of the residual thickness of the residual lining would be *x_p_* = 650 µm heat^−1^ then, using Formulas (4) and (5) for i = 14 and n = 14, new mean values of the unit wear rate and its standard deviation were calculated. The new *a posteriori* distribution is shown in Figure 8.

The predicted mean value of the unit wear rate and the standard deviation will be *μ_p_* = 418.6 µm heat^−1^ and σ_p_ = 13.1 µm heat^−1^, respectively. The calculated quantiles of the unit wear rate for the predicted *a posteriori* distribution calculated in µm·heat^−1^ are shown in Table 3.

**Figure 9:**
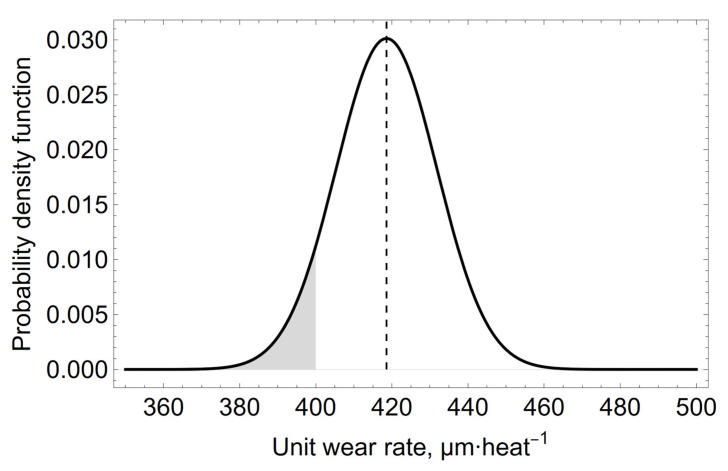
The shaded area shows an 8% probability that the value of the unit refractory wear rate in the oxygen converter slag spout zone will be below 400 µm heat^−1^.

A question that is important for converter users is, what is the probability for the specific wear rate to be, for example, less than 400 µm heat^−1^. After using a hypothetical 14th measurement of the specific wear rate (*x_p_* = 650 µm heat^−1^) in the calculation and determining the form of its *a posteriori* distribution, the probability that the specific wear rate will be less than 400 µm heat^−1^ is less than 8%, as shown in Figure 9 by the shaded area under the probability density distribution curve.

## 4. Discussion

The Bayesian evolution of the mean value of μ µm heat^−1^ unit wear rate of the refractory material MC 95/10 in the spout zone, shown in form of graphs in Figure 5, Figure 6 and Figure 7, illustrates the effect of changing the *a priori* distribution and the likelihood function on changing the *a posteriori* distribution by changing the mean value of the distribution and reducing its variance as the information from the measurement-sampling increases.

Subsequent iterations in the model calculations, carried out by increasing the sample amount (measurements), updated the knowledge of the distribution probability of the value of the unit refractory wear rate in the slag spout zone and its variance.

The results predicting the mean and distribution variance of the unit wear rate value of the steel converter’s refractory lining, obtained assuming the value *x_p_* = 650 µm heat^−1^ of the hypothetical 14th measurement, indicate a change in the parameters of the predicted probability distribution to the values of μ_p_ = 418.6 µm heat^−1^ and σ_p_ = 13.1 µm heat^−1^. The results shown in [4,5,6,7,8] cannot be directly compared with the above mentioned results, because they relate to other materials, devices, and research methodologies. The results of the measurements and calculations included in this article extend the range of data concerning the wear of refractory linings in oxygen converters.

The method of searching for an answer to the question of the probability of obtaining a value contained in a given range of values of the unit wear rate of a refractory lining is reduced to finding the size of the area under the curve of the forecast probability density distribution of the unit wear rate of a refractory material.

The choice of Bayesian statistics for forecasting the unit wear rate of MgO-C materials type MC 95/10 in the slag spout zone of an oxygen converter was dictated by the variety and complexity of factors affecting the decision to terminate the converter campaign. Making such a decision is conditioned, both by the laser measurement results of the residual refractory lining thickness of the converter, and by visual assessment of the condition of the converter’s refractory lining, which may vary depending on the operating experience of the people making the decision to stop the converter’s operation.

## 5. Conclusions

From the analysis of the carried out experiment and the calculations performed according to the rules of Bayesian statistics, the following conclusions are drawn:

From the experience gained during the long-term operation of the steel-producing oxygen converter, it was found that the value of the unit wear rate of the refractory material in the slag spout zone of the steel-producing oxygen converter is subject to *a posteriori* normal distribution, with the exemplary parameters: mean value µ= 401.23 µ heat^−1^, standard deviation σ = 13.74 µm heat^−1^.

The mean value of the unit wear rate during the oxygen converter’s campaigns between February and November 2019 was verified experimentally and was calculated as 420 µm heat^−1^, which is within the calculated confidence interval 375.7 ≤ µ_aposterior_ ≤ 428.2 µm heat^−1^.

The calculated results of the forecasted distribution of the unit wear rate value of the refractory material give valuable operational and cost information, which is important for steelmakers, as well as for refractory material manufacturers.

From a prediction point of view, of the value distribution of the refractory unit wear rate, it would be beneficial to distribute the successive measurements of the residual thickness of the refractory lining in the slag spout zone evenly over the course of the converter campaign.

This model allows the creation of “what if” scenarios, which can also be helpful in estimating the cost of a planned converter campaign.

## Figures and Tables

**Table 1:** Material properties of MC95/10.

MgO *	97.7 wt.%	XRF-X’Unique II
CaO *	1.5 wt.%	XRF-X’Unique II
SiO_2_ *	0.4 wt.%	XRF-X’Unique II
Fe_2_O_3_ *	0.3 wt.%	XRF-X’Unique II
Al_2_O_3_ *	0.1 wt.%	XRF-X’Unique II
Apparent porosity	1.5%	PN—EN 993-1
Bulk density	3.06 g·cm^−3^	PN—EN 993-1
Cold crushing strength	35 MPa	PN—ISO 10059-1
Total carbon content	14 wt.%	HFF—IR Leco CS300

*—share of the component in the magnesia part (without carbon and antioxidants).

**Table 2:** Selected quantile values for the unit refractory wear rate in the slag spout zone calculated from the a posteriori distribution.

Quantile	µ_aposteriori_, µm heat^−^^1^
0.01	369.2
0.025	374.2
0.05	378.5
0.25	391.9
0.5	401.1
0.75	410.4
0.95	423.7
0.975	428.1
0.99	433.1

**Table 3:** Selected quantile values for the predicted unit refractory wear rate in the slag spout zone for a hypothetical 14th measurement of *x_p_* = 650 µm heat^−1^, calculated from the a posteriori distribution.

Quantil	µ_aposterior,_ µm heat^−^^1^
0.01	387.8
0.025	392.6
0.05	396.8
0.25	409.7
0.5	418.6
0.75	427.5
0.95	440.4
0.975	444.6
0.99	449.4

## Data Availability

Not applicable.
